# Supplementary material for: Growth and DNA Methylation Alteration in Rice (Oryza sativa L.) in Response to Ozone Stress
Source: Genes (Basel). 2023 Sep 28;14(10):1888. doi: 10.3390/genes14101888 (PMC10606928; doi:10.3390/genes14101888)
Supplement: Supplementary file 1 [file genes-14-01888-s001.zip › genes-2610654-supplementary.pdf]

Table S1. Sequences of adaptors and primers for MSAP analysis.

**Primer Types**	**Primer Sequence（5'-3'）**	
	***EcoR* Ⅰ**	***Hapa* Ⅱ/*Msp* Ⅰ（H/M）**
Adaptor	CTCGTAGACTGCGTACC	GATCATGAGTCCTGCT
	AATTGGTACGCAGTC	CGAGCAGGACTCATGA
Primer	GACTGCGTACCAATTCA	ATCATGAGTCCTGCTCGG
Selective-amplification Primer	E-AAC	H/M-TCT
	GACTGCGTACCAATTCAAC	ATCATGAGTCCTGCTCGGTCT
	E-AAG	H/M-TCG
	GACTGCGTACCAATTCAAG	ATCATGAGTCCTGCTCGGTCG
	E-ACA	H/M-TCC
	GACTGCGTACCAATTCACA	ATCATGAGTCCTGCTCGGTCC
	E-ACT	H/M-TTC
	GACTGCGTACCAATTCACT	ATCATGAGTCCTGCTCGGTTC
	E-ACC	H/M-TTG
	GACTGCGTACCAATTCACC	ATCATGAGTCCTGCTCGGTTG
	E-ACG	H/M-TTA
	GACTGCGTACCAATTCACG	ATCATGAGTCCTGCTCGGTTA
	E-AGC	H/M-TGA
	GACTGCGTACCAATTCAGC	ATCATGAGTCCTGCTCGGTGA
	E-AGG	H/M-TGT
	GACTGCGTACCAATTCAGG	ATCATGAGTCCTGCTCGGTGT
	E-AGA	H/M-TGC
	GACTGCGTACCAATTCAGA	ATCATGAGTCCTGCTCGGTGC
	E-ATC	H/M-TAC
	GACTGCGTACCAATTCATC	ATCATGAGTCCTGCTCGGTAC
